# To Legislate or Not to Legislate? A Comparison of the UK and South African Approaches to the Development and Implementation of Salt Reduction Programs

**DOI:** 10.3390/nu6093672

**Published:** 2014-09-16

**Authors:** Karen Charlton, Jacqui Webster, Paul Kowal

**Affiliations:** 1 School of Medicine, University of Wollongong, Northfields Avenue, Wollongong, NSW 2522, Australia; 2 The George Institute for Global Health, Sydney, NSW 2050, Australia; 3 WHO Collaborating Centre on Population Salt Reduction, Food Policy Division, 83-117 Missenden Rd, Camperdown, Sydney, NSW 2050, Australia; E-Mail: jwebster@georgeinstitute.org.au; 4 WHO Study on global AGEing and adult health, 20 Avenue Appia, CH-1211 Geneva 27, Switzerland; E-Mail: kowalp@who.int; 5 Research Centre for Gender, Health and Ageing, University of Newcastle, University Drive, Newcastle, NSW 2308, Australia

**Keywords:** salt reduction, legislation, South Africa, United Kingdom, food policy, food supply

## Abstract

The World Health Organization promotes salt reduction as a best-buy strategy to reduce chronic diseases, and Member States have agreed to a 30% reduction target in mean population salt intake by 2025. Whilst the UK has made the most progress on salt reduction, South Africa was the first country to pass legislation for salt levels in a range of processed foods. This paper compares the process of developing salt reduction strategies in both countries and highlights lessons for other countries. Like the UK, the benefits of salt reduction were being debated in South Africa long before it became a policy priority. Whilst salt reduction was gaining a higher profile internationally, undoubtedly, local research to produce context-specific, domestic costs and outcome indicators for South Africa was crucial in influencing the decision to legislate. In the UK, strong government leadership and extensive advocacy activities initiated in the early 2000s have helped drive the voluntary uptake of salt targets by the food industry. It is too early to say which strategy will be most effective regarding reductions in population-level blood pressure. Robust monitoring and transparent mechanisms for holding the industry accountable will be key to continued progress in each of the countries.

## 1. Introduction

Globally, the prevalence and impact of hypertension and diets high in sodium increased between 1990 and 2010 [1]. The 2010 Global Burden of Disease ranks morbidity attributed to risk factors, with hypertension the leading factor and a high sodium diet ranked 11th as contributing to overall disability-adjusted life years (DALYs) [2,3,4]. In the USA, it has been estimated that a regulatory intervention designed to achieve a reduction in salt intake of three grams/day would save 194,000 to 392,000 quality-adjusted life-years (QALY), $10 to 24 billion in healthcare costs annually and be more cost-effective than using medications to lower BP in all persons with hypertension [5]. Against this backdrop and considering the ongoing demographic and epidemiological shifts in higher and lower income countries alike, salt reduction has been identified by the World Health Organization (WHO) as a “best buy” for public health efforts [6,7]. The 2012 WHO guidelines targeting daily salt intake of less than five grams are a sound starting point to reduce blood pressure and the risk of cardiovascular disease [8] and Member States have agreed to a 30% reduction target in mean population salt intake by 2025 [9].

The most recent estimates showed that in 2010, global mean salt intake was around 10 g per person per day, so around twice the WHO recommended amount. The East African Region had the lowest salt intake, at just over five grams per person per day, while the Central East Asia Region had the highest at around 13 g [10]. An increasing number of countries around the world are adopting population-based salt reduction strategies. Many countries that have implemented programs to date have been countries where a high proportion of salt in the diet comes from processed foods. It follows then that one of the main strategies has been to encourage the food industry to voluntarily reformulate products to be lower in sodium [11]. The United Kingdom (UK) and South Africa are two countries that have considerably different population structures, mean life expectancies and disease burden profiles, but are nonetheless both working to reduce salt through food industry reformulation [12,13]. Most recent estimates calculate salt intake to be 8.6 g/day for the UK [14] and between 7.8 and 9.5 g/day for South Africa, depending on ethnic group [15]. The UK provides the most comprehensive and successful example of the development of voluntary targets for salt levels in different food product categories, and many other countries have followed suit. However, there is a growing trend towards legislation, with maximum salt levels being established for bread in a number of countries, including Belgium, Greece, Hungary, The Netherlands, Portugal and Paraguay [16,17]. Bulgaria has extended the legislation to bread, milk products and *lutenica* (a vegetable relish), and Argentina has legislated salt levels for a range of products, including bread and processed meats [16,17].

The South African government recently adopted a more comprehensive legislative approach by passing legislation for salt levels in a wide range of processed foods. The initial salt level standards will become mandatory as of 30 June 2016, with more stringent maximum levels coming into place from 30 June 2019 [18].

This article provides a review of the historical processes, the establishment of the evidence bases and the subsequent advocacy and lobbying in the UK and South Africa that led to the adoption of their different salt reduction strategies. Narrative documentation of the author’s own experiences, supplemented through relevant scientific papers, campaign materials and evaluations, as well as consultation responses, provided the basis for a comparative analysis of UK and South African approaches.

## 2. The UK’s Voluntary Approach to Salt Reduction

### 2.1. Overview of the United Kingdom’s Salt Reduction Target Process

Prior to the UK government’s Food Standards Agency (FSA) making salt reduction a public health priority in 2003, debates about salt between government, the food industry, the Salt Manufacturer’s Association and health advocacy groups had been running for decades [19,20]. The 1974 COMA (Committee on the Medical Aspects of food policy) report acknowledged the evidence, and even in the mid-1970s, many in the food industry were reportedly prepared for action by the government to mandate salt reduction, while table salt sales had already begun to fall [20]. However, subsequent government reports [21] resulted in sustained industry-funded publicity questioning the evidence base linking salt to ill health and promoting salt [20]. This included the creation of a “Salt Data Centre”, which was touted as an independent centre, but which really functioned solely to dispute the relationship between salt and blood pressure [22,23].

Subsequent government reports made no strong recommendations until the early 1990s, when dietary targets for salt were set and the government made clear recommendations for reductions in the sodium levels of manufactured foods [24,25,26]. The government later backtracked, however, as industry representatives baulked, and the task force set up to deal with the issue was disbanded [27,28]. Health advocacy groups organized themselves and began to collaborate. The Consensus Action on Salt and Health (CASH) group was established in 1996 and immediately began a sustained effort to counter some of the lobbying activities of the food industry. This included publishing comprehensive reviews of available evidence in respected scientific journals [22], regular monthly surveys of salt levels in foods supported by high profile media campaigns and organising briefing sessions for incoming government ministers. Support for the industry opposition waned as progressively more conclusive evidence was accumulated and disseminated [29,30,31].

Following a change in the UK government in 1997, salt reduction was once again placed on the agenda, and a further review of the evidence was commissioned by the newly established Food Standards Agency. The Scientific Advisory Committee on Nutrition (SACN)’s report on Salt and Health, published in June, 2003, provided a comprehensive up-to-date overview of the research evidence and confirmed the need for action, stating that, “Key to achieving a sustained salt reduction for public health benefit is the engagement of the food industry” [32]. This report became the platform for the development of the current UK Government salt reduction strategy. The strategy was officially launched by the FSA in 2003, almost three decades after the original COMA report. Despite previous opposition, the food industry has been a key partner and continues to support the campaign [33].

#### 2.1.1. Development of the Salt Model

It was estimated that 15% of salt consumed in the UK was added during cooking and at the table and that 5% was naturally occurring in foods, with the remaining 80% coming from processed foods [34]. Existing dietary survey and food composition data were used to develop a salt model, which highlighted the percentage contribution of different processed foods to salt intake [34]. The model was based on the fact that for adults aged 19–64 years, white bread contributed 10.3% to salt intake, bacon and ham 8.1%, breakfast cereals 4.9% and homemade meat-based dishes 4.4%. Cheese, sausages, fat spreads, baked beans and milk and cream contributed between 3% and 4% each. The food categories of other bread, wholemeal bread, soup, pizza, crisps/savoury snacks and meat pies each contributed between 2% and 3% to the total non-discretionary salt intakes.

This salt model was used to calculate how much salt would need to be removed in each food category to reduce the salt content of the food supply by 40%, which (if combined with a campaign to reduce the amount of salt added during cooking and at the table by a similar amount) would move average population intake from 9.5 g of salt/day to 6 g of salt/day. It was acknowledged that very large reductions in some of the main product categories would be difficult to achieve; therefore, the model highlighted the need for a broad-based approach that sought to achieve some reduction in almost all food categories.

The salt model was the first step towards establishing category-specific targets in the country and was used as the basis for negotiating actual targets with the food industry, taking into account both the technical feasibility related to food safety and processing mechanisms, as well as consumer acceptability of reduced salt foods. The salt model was published for public consultation in 2003, and at the same time, a broad program of food industry engagement was instigated.

#### 2.1.2. Engaging the Food Industry

**Figure 1:**
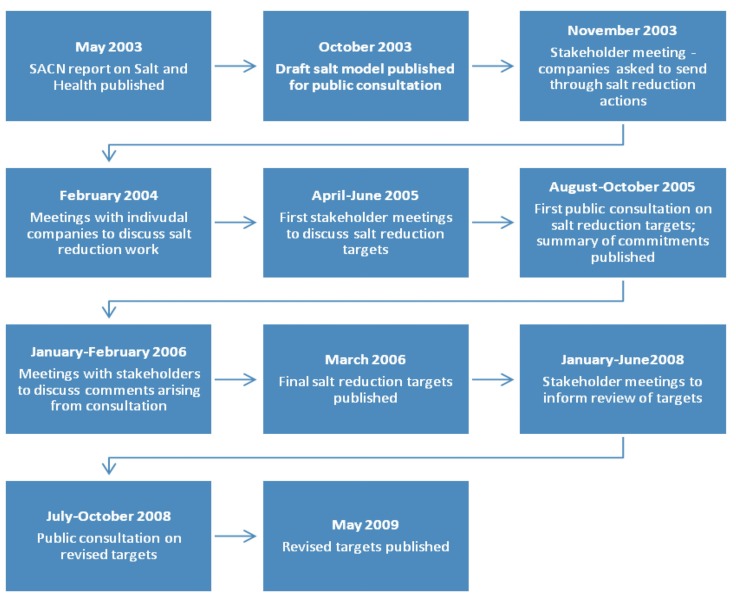
Summary of the UK Food Standards Agency’s engagement with the food industry to develop salt reduction targets, 2003 to 2009. SACN, Scientific Advisory Committee on Nutrition.

The approach of the UK FSA in engaging the food industry has been well documented [34] (see Figure 1).

#### 2.1.3. Stakeholder Forum and Industry Consultation

Following the publication of the SACN report in June, 2003, a high-profile stakeholder forum was convened jointly between the FSA and the Department of Health in November, 2003 [35]. The forum was jointly chaired by the Health Minister and the Chairman of the Food Standards Agency and attended by senior civil servants, industry CEOs, academics and health and consumer organisations. The objective was to review the recommendations made in the SACN report and to consider actions that could be taken to address national salt consumption patterns. The government made a commitment to work towards reducing salt intakes in line with the report’s recommendations and developed a program of work to achieve that goal, with three main targets: (1) a public campaign to raise consumers’ awareness of why a high salt diet is bad for health and what can be done to reduce intake and risk; (2) a program to work with the food industry to reduce salt levels in foods; and (3) front-of-pack labelling to provide additional information to consumers on the levels of salt (and other nutrients) in food [36].

The different sectors of the food industry were engaged in a number of ways, including through written consultations, working groups with representatives from multiple companies and face-to-face meetings with individual companies. Individual company commitments and action plans were negotiated and published on the agency website. Trade associations were used to communicate issues to a range of companies or a particular sector. Retailers, for example, were engaged through the British Retail Consortium and food manufacturers through the Food and Drink Federation. Separate strategies were developed for communicating to small businesses and the catering sector to ensure that they had the required information and technical support to make reductions in parallel with the market leaders [37].

#### 2.1.4. Cross-Sector Collaborations

Cross-industry collaborative groups were established on bread, breakfast cereals and soups and sauces to encourage corporate collaboration between companies and to agree on commitments to salt reductions in their respective products. For example, the bread and breakfast cereal manufacturers initially agreed to work towards annual reductions of 10% for three consecutive years from 2003. Many of these manufacturers have since continued making reductions beyond the initial target [38]. A similar initiative, Project Neptune, was set up for soups and sauces and operated over the same timeframe [39]. Together, the companies and trade associations set up systems for monitoring their own progress towards these commitments. This helped to ensure that there was a level playing field across specific product categories and that companies made reductions simultaneously to avoid the danger of customers switching from one brand to another if they perceived a difference in flavour.

#### 2.1.5. Developing Targets for Salt Levels in Foods

Once industry stakeholder engagement had been established, targets for maximum levels of salt in each food category were developed, taking into account technical, safety and consumer acceptability issues. The targets were designed to be challenging enough to ensure that they would impact on population salt intake and population health. At the same time, they were set at a level that had already been achieved by the lower salt products in the category, demonstrating technical feasibility. Progress towards achieving the targets was monitored through both industry self-report and the establishment of a database of salt levels in the top food categories that contribute to salt in the diet [40]. In 2009, many of the major retailers and manufacturers reported that they had achieved the 2010 targets; therefore, more challenging new targets were set, to be met by 2012 [36]. In 2011, however, political changes threatened the ongoing success of the salt reduction campaign. A newly appointed coalition government handed back the nutrition activities of the FSA to the Department of Health. UK salt experts feared that the salt reduction campaign would no longer be supported and have claimed that the change in government resulted in slowing of the momentum for three years [12]. Rather than being government led, the new Public Health Responsibility Deal was based on the premise of collective action, which many feared would undermine progress. However, three salt reduction pledges, including new pledges on catering and home meals, as well as revised 2017 targets for 72 categories of processed foods have so far been announced through the Responsibility Deal’s Food Network [41].

#### 2.1.6. Impact of the UK Salt Reduction Campaign

The UK salt reduction strategy is multi-faceted, comprising work with industry, as well as a social marketing campaign to change consumer behaviour, accompanied by clearer food packaging labelling of salt content. At the outset of the campaign, the government promised the food industry that in return for their efforts to reduce salt in foods, the FSA would launch a high profile campaign to inform consumers that salt was bad for health and that they needed to try and reduce salt in their diets, thus driving consumer demand for the newly reformulated reduced salt foods. At first, food companies did not want to alert consumers to the fact that salt was being reduced in their products, for fear of consumer rejection of the reformulated versions. Salt was therefore reduced gradually and in silence.

Meanwhile, the FSA launched its famous “Sid the Slug” campaign to raise awareness that salt was bad for health. The multimedia campaign included advertising billboards, television commercials and internet coverage and was based on the premise that salt kills slugs, and can harm humans, too. The campaign infuriated the Salt Manufacturers’ Association (SMA), who complained to the Advertising Standards Authority that the information was misleading. Following an extensive review of the evidence base, the Advertising Standards Authority rejected the SMA complaint in its adjudication. Ironically, the controversy resulted in extensive media coverage, which added considerably to the reach of the campaign, and at no extra cost. Evaluation after the first stage of the campaign revealed that within a year, the proportion of people that knew the dietary target for salt had increased from 3% to 34% [42].

The second stage of the FSA awareness campaign focused on behaviour change. The slogan, “Is your food full of it?” was launched, together with a series of targeted TV advertisements and other campaign materials to encourage consumers to check food labels and select products with the lowest salt content. At this stage, the food industry started to recognize that people were becoming increasingly interested in consuming a low salt diet. One multinational company supported the FSA campaign by including the slash “Check out our salt levels” on the front of its premium brand products, whilst an upmarket supermarket chain displayed in-store posters saying, “We’re reducing salt in our foods faster than you can say sodium chloride”. For the first time in decades, the government, health advocacy groups and, now, even the food industry were presenting a united salt reduction message. Subsequent evaluation of the social marketing campaign has demonstrated an impressive impact in terms of reported changes in consumer behaviour [43].

The campaign reported a reduction of about a gram of salt per person per day in 2008 [34]. By 2010, most processed foods available in supermarkets had salt levels that were 20%–30% [43,44] lower than when the process commenced in 2003. The UK has since reported further reductions in salt intake, totalling around 15%. This level of reduction is estimated to save approximately 8000 lives annually [45]. Even more recent work has demonstrated parallel reductions in blood pressure and stroke mortality, which are highly likely to be due to population-level reductions in salt intake [14].

### 2.2. South Africa’s Legislative Approach to Salt Reduction

In 2012, South Africa established a daily salt intake target of less than five grams per person by 2020. However, like the UK, the benefits of salt reduction were being debated in South Africa long before it became a policy priority. Whilst salt reduction was gaining a higher profile on the international policy agenda, the availability of local, context-specific, domestic costs and outcome indicators proved crucial to influencing the legislation. Research conducted in the mid-2000s followed by extensive academic engagement with the South African Government and non-governmental organizations, such as the Heart and Stroke Foundation, led to the introduction of government policy directives on salt reduction.

The South African Medical Research Council (MRC) produced a policy brief [46], which summarised the body of research that, importantly, demonstrated the feasibility of the proposed strategy to reduce salt in the South African food supply [47]. Modelling showed that a reduction in the sodium content of bread by 50%, along with other proposed reductions in margarine, soups and gravies, would decrease salt intake by 0.85 grams per day, resulting in 7000 fewer deaths due to cardiovascular disease and 4000 less non-fatal strokes in the country per year. The estimated cost savings related to this level of salt reduction is 300 million Rands (~US$30 million) each year in healthcare costs associated with non-fatal strokes alone [48].

The legislative process in South Africa came about through international political pressure coinciding with new local evidence and embraced by political opportunism. In September, 2011, influential leaders gathering at a United Nations high-level meeting on non-communicable diseases (NCD) in New York reached consensus on the global priority action needed to prevent and treat these conditions [4] and identified salt reduction as one of five overarching priorities for action. Ahead of that meeting, an NCD summit that was held in Johannesburg (September 12–13, 2011) resulted in Aaron Motsoaledi, Minister of Health for South Africa, making a commitment to reduce mean population salt intake, including through regulation of the food industry. Previous engagement and lobbying by academics and national and international public health advocates to the Ministry of Health had meant that existing scientific evidence was available to support the introduction of a salt reduction strategy. Thereafter, academics and international experts were invited to a series of government-industry consultations, which began with the baking industry, but were expanded to a range of other food processing industries [13]. Voluntary salt targets set by other countries, including the UK, Australia and USA, were consulted to ascertain the feasibility of target levels of salt across food categories. This led to the development of draft legislation for permitted salt levels in a variety of processed foods, which were published on 11 July 2012 [49].

#### 2.2.1. Estimating Salt Intake Levels

Research to understand the contributions of different foods to salt intakes in South Africa informed the legislation. Repeated 24-h urinary collections in different population groups provided estimates of usual total sodium intake. A study in Cape Town reported ethnic differences in average daily salt intakes, equating to 7.8, 8.5 and 9.5 g/day in black, mixed ancestry and white individuals, respectively [15]. Dietary surveys were used to understand sources of salt in the diet. Using a crude approach based on the difference between reported dietary intake and urinary sodium excretion, it was assumed that up to 46% of the salt consumed by South Africans comprises discretionary salt added during preparation of food or at the table.

#### 2.2.2. Identifying Contribution of Different Processed Foods to Salt in the Diet

Having established that 54% of the salt in the diet came from processed foods, the next step was to identify which other foods were contributing to salt in the diet. The diverse ethnic groups in South Africa have markedly different eating patterns and are at different stages in the nutrition transition (characterised as a change in diet from a traditional high carbohydrate, high fibre, low fat diet to one with a higher fat and sugar intake and a lower carbohydrate and fibre intake [50]). These differences necessitated a dietary survey approach that was able to identify food consumption patterns specific to cultural groups.

**Figure 2:**
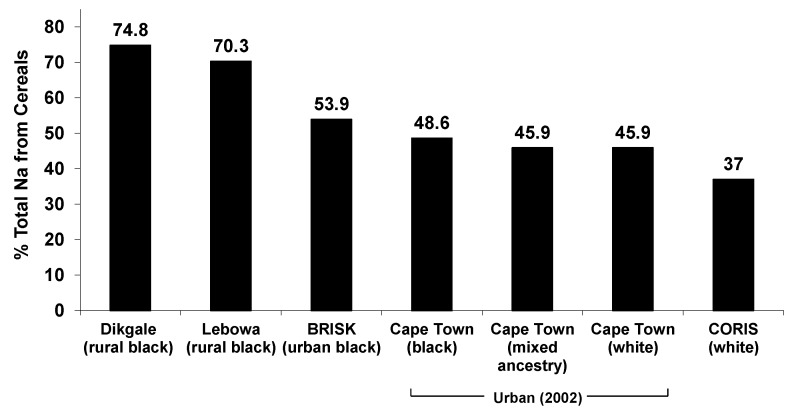
The contribution of the bread and cereals food group to total non-discretionary sodium intake in surveys of South African adults, by ethnic group (this figure is reprinted from Charlton, *et al*., 2005 [56]. Copyright 2005, with permission from Elsevier Inc.).

At that time, there were no nationally representative dietary studies that had been conducted in South African adults. A secondary analysis of four dietary surveys that used a 24-h recall method was undertaken to assess quantities and food sources of sodium intake. This included two studies of rural black subjects [51,52,53,54], a study of urban black residents in Cape Town (Black Risk Factor Study; BRISK) [52] and a study of rural white subjects in the Western Cape (The Coronary Risk Factor Study; CORIS) [55]. In all of these surveys, the bread and cereals food group was the largest contributor to total sodium intake, ranging from 37% in white rural subjects to 73.1% of total salt intake in rural Africans (Figure 2). In the two studies of rural black South Africans, a much higher contribution of this food group was evident (70.3%–74.8%) compared to city-dwelling black Africans (45.9%–53.9%) [53].

A fifth study that included three repeated 24-h dietary recalls in a multi-ethnic sample in Cape Town also helped to identify sources of salt in the diet [56]. As well as bread, other important food sources of salt included meat products (*boerewors*, a seasoned beef sausage, meat pies, processed meats, such as polony, vienna, salami, ham and other sausages), as well as soup powders and brick margarine. The practice of adding monosodium glutamate-based flavour enhancers (for example, Aromat and Fondor) and stock cubes to staple foods (maize meal) during preparation is common in South Africa, particularly in disadvantaged communities, and contributes to the high sodium intake in the black, majority population. The relative contributions of these different foods by different ethnic groups are summarized in Table 1.

**Table 1:** Contribution of top 20 individual food items to total non-discretionary sodium intake ^a^, in a multi-ethnic sample in Cape Town, South Africa (this table is redrawn from Charlton, *et al*., 2005 [56]. Copyright 2005, with permission from Elsevier Inc.).

Rank	Food item	% Total Na^+^ intake		
		Black *n* = 110	Mixed ancestry *n* = 112	White *n* = 103
	Bread, all types	40.54	30.70	25.18
1	Bread/rolls, white	22.26	22.03	15.24
2	Bread/rolls, brown	17.27	6.68	5.74
	Bread/rolls, whole wheat	1.01	1.99	4.20
3	Beef sausage, *boerewors*	4.15	6.62	2.43
4	Steak and kidney pie (commercial)	3.42	1.29	1.70
5	Soup powder (reconstituted)	2.93	-	-
6	Margarine, brick/hard	2.90	1.89	1.51
7	Polony	2.53	2.17	-
8	Maas/sour milk	2.44	-	-
9	Potato chips/French fries	2.21	1.65	1.55
10	Milk, full cream, fresh	2.12	1.90	1.56
11	Potato crisps	1.96	2.73	1.30
12	Popcorn, plain	1.41	-	-
13	Salami, pork/beef (Russian)	1.38	0.97	-
14	Sausage roll (commercial)	1.38	0.91	0.91
15	Breakfast cereal, all-bran flakes	1.22	1.51	4.19
16	Soup, vegetable (canned)	1.19	-	1.51
17	Vienna sausage (canned)	1.14	1.14	2.55
18	Chicken pie (commercial)	1.13	1.13	-
19	Aromat	1.13	-	-
20 ^b^	Breakfast cereal, corn flakes	-	2.90	3.06
	Cheese, cheddar		1.92	1.88
	Savoury snack, corn chips	-	1.53	-
	Fish biltong (salted, dried cod)	-	1.19	-
	Baked beans	-	1.01	-
	Sausage, pork	-	-	1.93
	Pizza	-	-	1.76
	Ham (cooked/canned)	-	-	1.32
	ProVita crackers	-	-	1.30
	Bacon fried, lean	-	-	1.27
	Low fat spread, polyunsaturated	-	-	1.19

^a^ Arranged in descending order of % total Na^+^ intake (group); ^b^ Foods not number ranked after 20, as the ranking relates to the top 20 foods identified in the black sub-group. Other ethnic groups had different rankings; foods contributing to the top 20 in those groups are included.

#### 2.2.3. Cross-Sector Collaborative Research (Industry-Government-Academia)

**Figure 3:**
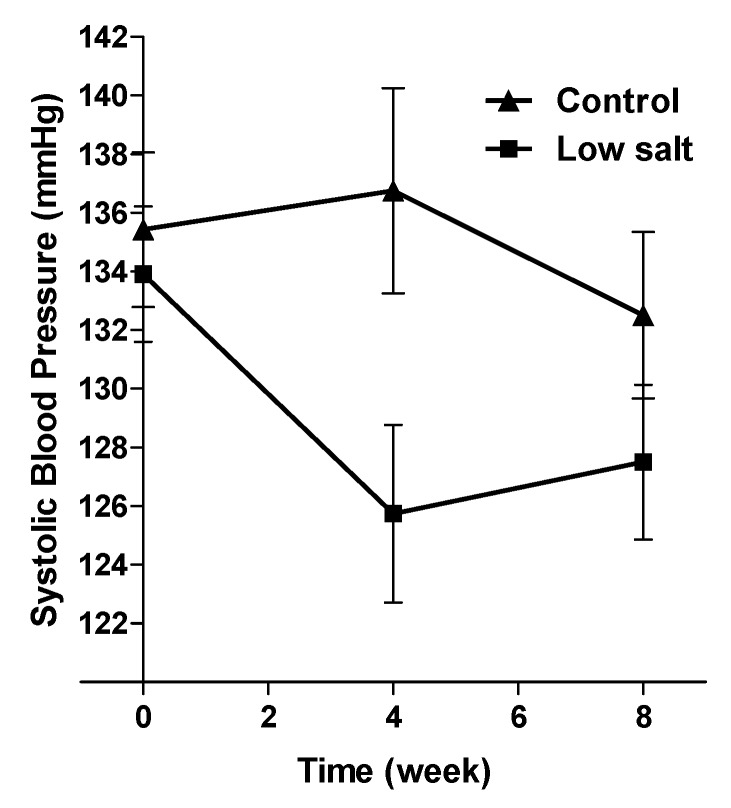
Mean systolic blood pressure change in an eight-week RCT that provided reduced sodium variants of commonly consumed food products to hypertensive South Africans (confidence intervals reflect the SEM) (this figure is redrawn from Charlton, *et al.*, 2008 [47], Copyright 2008, with permission from Cambridge University Press).

Once evidence about food sources of salt was available, researchers worked with food industry partners that included R & D staff, food technologists, consumer affairs and marketing managers to develop sodium-reduced variants of bread [57], margarine, stock cubes, flavour enhancers and soup mixes, to ensure the technological properties and consumer acceptability [58]. Cost analysis identified that replacement of salt in bread with a commercial salt replacement (SOLO Low Sodium sea salt™, a salt replacement produced from Icelandic water, which provides 60% less sodium than ordinary table salt) that contains both magnesium and potassium would add 30 cents per loaf (at the 2004 cost of raw materials), compared to 8.9 cents per loaf for replacement with salt mixes that were developed in the Research and Development (R & D) laboratory of the country’s third largest bread producer. These experimental products, which were commercially produced in existing factory lines, were used in a food-based, eight-week, randomized, controlled trial to assess the magnitude of the blood pressure reduction of substitution with salt-reduced variants of these few food products [47]. Importantly, the study was conducted in a resource-poor community setting, which demonstrated that the substitution of a limited number of commonly consumed food items, in the presence of the daily consumption of 500 mL of fermented milk product (*maas*) and the substitution of table salt with a salt substitute (SOLO™) lowered blood pressure by a clinically significant magnitude in community-dwelling, hypertensive patients (Figure 3). The magnitude of the BP-lowering effect (−6.2 mm Hg (95% CI 0.9, 11.4) for systolic BP; *p* = 0.021) was similar to that shown by the use of diuretic therapy. However, additional benefits were observed, over and above standard pharmacotherapeutic approaches, as all participants had a diagnosis of hypertension and were receiving standard care according to primary care clinical guidelines. The outcomes of the study showed that many of the beneficial nutrients obtained through the adoption of a DASH (Dietary Approaches to Stop Hypertension) diet could be obtained simply by modifying existing commonly consumed food items to contain higher amounts of potassium and magnesium and lower amounts of sodium [59,60].

#### 2.2.4. Establishing the Maximum Salt Levels for the Legislation

Final decisions about the maximum levels of salt to be allowed in foods were driven by the food industry-partnered research on the development of reduced sodium bread [57], as well as the research team’s experience of consumer-tested sodium-reduced variants of stock cubes, soup powders, brick margarine and flavourants that were used in an experimental RCT [58].

Publication of the draft regulations was followed by a three-month open public consultation process. After review, the Ministry made adjustments to timelines and adjusted the salt content of some of the food categories, as published in the final government gazette dated 20 March 2013 [18], and shown in Table 2. During the consultation process, some sectors of the food industry voiced their opposition to the legislation. Members of the baking industry expressed concern that the originally proposed 2019 threshold of 370 mg Na/100 g for bread would change the taste, texture and bread-making process to such an extent that the industry would need to invest in new ingredients and machinery [61]. The final legislation relaxed this target to 380 mg Na/100 g bread (Table 2). Of note in the debate about bread is that the UK salt target for bread is 360 mg Na/100 g bread for the average across all ranges, and a maximum of 450 mg Na/100 g bread (Table 2). Publication of the draft regulations was widely covered by the media, which provided opportunities to disseminate information and increase public awareness of the association between salt and hypertension and the extent of the burden of disease.

It is notable that many of the maximum permitted levels of salt allowed in food categories are much higher than the current UK targets, particularly for processed meats, savoury snacks, such as crisps, and breakfast cereals. It is difficult to compare targets for dried soup powders and stock cubes, as the UK targets specify levels of salt in the products as consumed. Due to the common practice of using these products as condiments to flavour staple maize meal dishes in South Africa, the South African targets report levels as purchased in the dry form. The more stringent targets that are stipulated for 2018 for these dry goods reflect a period of adaptation for consumer taste preferences.

A further difference between the South African and the UK targets is that the UK has established both maximum levels, which all companies are encouraged to try and achieve, as well as average levels, which are usually much lower and which represent the average for the product category as a whole. The idea is to reflect the often wide variations of salt levels in products, even within a specific product category, and still ensure that levels are reduced by as much as possible. In addition, monitoring of salt levels is also weighted by sales to account for the fact that even relatively small reductions in salt levels in products with a large share of the market will have a significant impact on population salt intake levels. Conversely, large reductions in salt levels in products that have a small share of the market will have limited impact. Whilst the combination of maximum and average targets is likely to be a more effective approach, the challenges associated with monitoring against average targets means that many countries prefer to stipulate maximum levels.

#### 2.2.5. Impact of the South Africa Salt Reduction Campaign

It is obviously too early to assess any impact of the South African salt reduction strategy. The fact that a large proportion of salt in the diet is added during cooking and at table meant that, in addition to working with the food industry to reduce salt in processed foods, any salt reduction strategy in South Africa would also need an accompanying social marketing campaign to target changes in individual consumer behaviours. Ahead of the legislated targets for salt reduction coming into place in mid-2016 and mid-2018, an advocacy group called SaltWatch has been established to complement government legislation by developing a national salt awareness campaign [62], which mirrors the approach taken in the UK SaltWatch was launched in Johannesburg on 13 March 2014 [63], and has received funding from the national Department of Health to run a six-month mass media campaign that will begin in late July, 2014. The campaign will make use of free-to-air television channels, as well as radio broadcasting to reach its target audience of women aged between 18 and 35 years, in Living Standards Measure categories 3–7. In the meantime, anecdotal evidence suggests that the food industry seems to be acting ahead of the target dates by voluntarily implementing salt reduction strategies in their food supply chains. The early adopters include many of the large, trans-national food companies that operate in South Africa and that have social responsibility clauses related to healthier reformulation of their product ranges, including salt reduction.

The same evidence-based approach that was used to establish the legislation is now warranted for the monitoring and evaluation of the effectiveness of the legislation. Whilst the efforts of advocacy groups, such as SaltWatch, are important, government funding also needs to be allocated to ensure that robust monitoring mechanisms are in place by the time the legislation comes into effect in June, 2016. This includes monitoring of the salt content of foods and shifts in population-level salt intake and blood pressure distribution, as well as evaluation of compliance of the food industry sector with the salt targets and the impact of the consumer awareness campaign.

In the meantime, one proposal for monitoring changes in salt intake and blood pressure is to leverage data from existing surveys and to include measures of 24-h urinary sodium excretion in these surveys. An example is the multi-country WHO Study on global AGEing and adult health (SAGE) in South Africa, a cohort study that collects comprehensive longitudinal information on the health and well-being of adult populations and the ageing process [64]. The survey comprises a nationally representative sample of older adults aged 50-plus years in South Africa *n* = 3842, as well as a smaller comparative sample of 385 individuals aged 18–49 year. Wave 0 was completed in 2004 and Wave 1 in 2008. Wave 2 is currently interviewing respondents, and Wave 3 is planned for 2016. Wave 2 data collection will include 24-h urinary sodium analyses in a sub-sample in order to provide representative baseline measures of salt intake against which to monitor the progress and effectiveness of the new South African legislation. The SAGE study is also taking place in Ghana, which will provide a comparative African country that does not have a policy to influence the sodium levels in the food environment, but that has similar levels of hypertension and increasing obesity and which can therefore act as a control to the South African study. Additionally, all six SAGE countries will include a set of standard questions on salt behaviours [65], providing information about between- and within-country trends over time.

**Figure 4:**
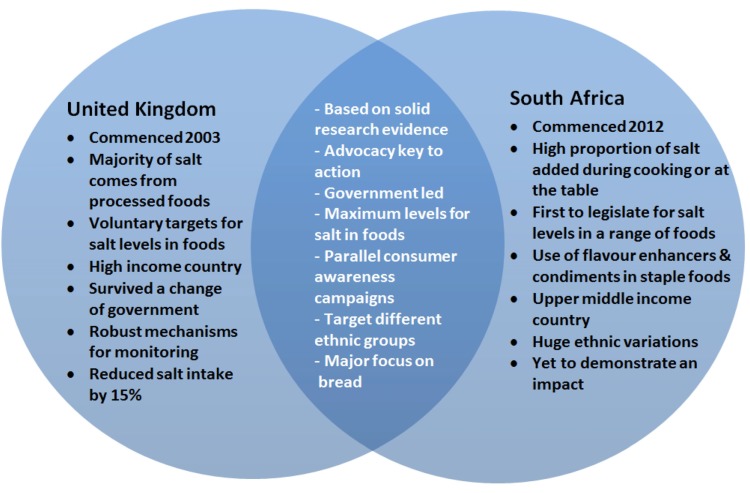
Venn diagram to show the similarities (areas of overlap) and differences between the UK and South African salt reduction strategies.

**Table 2:** Comparison of main salt level targets for processed foods in the UK and South Africa.

South Africa food category		South Africa maximum sodium (mg) per 100 g foodstuff by 30 June 2016 and 2019 [18]	UK voluntary average and/or maximum sodium (mg) target per 100 g by 2017 [66]
Bread		400 (2016) 380 (2019)	360 (average *r*) 450 (maximum)
All breakfast cereals and porridges, whether ready-to-eat, instant or cook up, hot or cold		500 (2016) 400 (2019)	235 (average *r*) 400 (maximum)
Spreads	Fat spread	550 (2016) 450 (2019)	425 (average *r*) 550 (maximum)
	Butter spread	550 (2016) 450 (2019)	Salted butters: 590 (average *r*) 670 (maximum) Lightly salted butters: 450 (average *p*)
Ready-to-eat savoury snacks, excluding salt-and-vinegar flavoured savoury snacks		800 (2016) 700 (2019)	Extruded and sheeted snacks: 680 (average *r*) 800 (maximum)
Flavoured potato crisps, excluding salt-and-vinegar flavoured potato crisps		650 (2016) 550 (2019)	525 (average *r*) 580 (maximum)
Flavoured, ready-to-eat, savoury snacks and potato crisps salt-and-vinegar only		1000 (2016) 850 (2019)	750 (average *r*) 1000 (maximum)
Processed meat (Classes 6, 12 or 14 of the South African National Standard SANS 885:2011), uncured		850 (2016) 650 (2019)	Cooked uncured meat—whole muscle: 270 (maximum) Reformed whole muscle: 360 (maximum) Comminuted or chopped reformed meat: 540 (maximum) Burger and grill steaks: 300 (average *r*) 350 (maximum) Frankfurters, canned hot dogs, canned burgers: 550 (average *r*) 700 (maximum) Fresh chilled frankfurters: 600 (average *r*) 750 (maximum)
Processed meat (Classes 6, 12 or 14 of the South African National Standard SANS 885:2011), cured		950 (2016) 850 (2019)	Ham/other cured meats: 650 (average *p*)
Raw-processed meat sausages (all types) and similar products		800 (2016) 600 (2019)	Sausages—all fresh, chilled or frozen: 450 (average *r*) 550 (maximum) Cooked sausage and sausage meat products: 550 (average *r*) 680 (maximum)
Dry soup powder (not the instant type)		5500 (2016) 3500 (2019)	Wet and dried soups as consumed 210 (average *r*) 250 (maximum)
Dry gravy powders and dry instant savoury sauces		3500 (2016) 1500 (2019)	All gravy as consumed: 380 (average *r*) 450 (maximum)
Dry savoury powders with dry instant noodles to be mixed with a liquid		1500 (2016) 800 (2019)	Noodles, plain and flavoured as consumed: 200 (average *r*) 350 (maximum)
Stock cubes, stock powders, stock granules, stock emulsions, stock pastes or stock jellies		18000 (2016) 13000 (2019)	Stocks as consumed: 300 (average *r*) 380 (maximum)

Notes: Average *r*, average used to account for a range of different flavours (including, potato crisps) or products covered by a single target; Average *p*, processing average used to account for ranges of salt levels that occur in a single product, for example, bacon and tuna. All range averages should be calculated on a sales weighted basis.

## 3. Discussion

Hypertension, or high blood pressure, is a common risk factor for stroke, coronary heart disease and kidney disease and is the leading preventable risk factor for death in the world [66,67]. There is an extensive body of evidence that demonstrates a strong association between salt intake and blood pressure [4,31,68,69,70,71,72]. Given that the WHO Global Action Plan for the Prevention of NCDs (2013–2020) includes a target for a 30% reduction of population-level salt intake by 2025, there is an urgent need to identify the optimal strategies to reduce population salt intake [73]. The UK was the first country to take leadership in target setting for food composition with voluntary salt targets agreed for around 80 categories of processed foods in 2006. This approach has now been adapted or adopted by a range of other countries. However, many public health experts argue that legislation is required [74], and in 2012, South Africa became the first country to introduce legislation on salt reduction in a wide range of processed foods, to be implemented in mid-2016. Whilst it is too early to confirm whether or not legislation is more effective than voluntary salt targets, the following insights have been gained from examining the similarities and differences between the two approaches (See Figure 4).

In terms of similarities, both strategies were initiated as a result of new or updated, culturally appropriate evidence. The comprehensive review and update of salt reduction evidence by the Scientific Advisory Committee on Nutrition in the UK was an important tool to galvanise the political support required for the UK to launch its salt reduction strategy in 2003. This confirmed that existing evidence was strong enough to necessitate action and was translated into a consumer-friendly format to help obtain public support. Likewise, experience from our review of the South African experience has also clearly demonstrated that original data [13,15,46,47,48,57,58] on salt intake patterns and cultural practices related to salt use were required to influence policy direction and lobby for legislative changes in the food supply in South Africa, leading to the new legislation in 2012.

Another striking similarity is the importance of continued advocacy by academics and/or non-governmental organisations to influence policy agendas. CASH in the UK was a key driving factor for government action on salt reduction in that country, while academics in South Africa have played an important role in persuading the government to act by strengthening the evidence and highlighting the cost effectiveness of taking action. Once salt reduction strategies are in place, academics and advocacy organisations need to continue their activities to ensure independent monitoring of the implementation and hold both government and the food industry to account.

In both the UK and South Africa, the action taken by government was fairly bold. The UK was the first country to launch a comprehensive national salt reduction strategy in the face of continued industry-fuelled controversy over the evidence. One of the drivers for this in the UK was the establishment of the Food Standards Agency, which was at arms-length from government. However, with a remit to protect the interests of consumers in relation to food, this Agency had the capacity and political independence to take on the salt reduction campaign in a much more targeted and dynamic way than typically pursued by government health departments.

Another similarity between the UK and South Africa is the adoption of multi-pronged approaches to reduce population salt intake. Population-based approaches to disease reduction need inter-sectoral collaboration, underpinned by stronger leadership from policy makers, advocates and health professionals. The pragmatic CVD-Risk Management package developed by the World Health Organization (WHO) to facilitate cardiovascular risk assessment and management in low-resource settings [75,76] provides guidance for cost-effective health services-based interventions. However, success depends on the capacity of primary healthcare systems to deliver these interventions and serve the long-term needs of high-risk CVD patients. For many countries, including South Africa, the individual management of large numbers of patients with low CVD risk is simply not affordable. As elucidated by the epidemiologist Geoffery Rose [77], it is individuals at lower risk, and not those at high risk, who account for a greater share of the overall disease burden. The management and prevention of CVD needs to be shifted to population-wide strategies that address major CVD risk factors and salt reduction strategies are a good example of this [78].

The effectiveness of salt reduction strategies is based on their potential to change the food environment. Such an approach requires not only buy-in from the food industry to reformulate food products, but commitment from government to support co-ordinated programs to change consumer behaviour. This is particularly true for South Africa where, in contrast to the UK, a high proportion of salt consumed still comes from salt added during cooking or at the table. Behaviour change programs can include the promotion of food-based dietary guidelines [79], but need to be supported by broad-based social marketing campaigns. The multi-sectoral coalition, which includes the National Department of Health and the Heart and Stroke Foundation South Africa, and which has just launched the SaltWatch mass media campaign in South Africa, is well positioned to take on this role [63]. The UK can provide important lessons in this regard, as it has over a decade of experience with such activities.

Clearly the main difference between the UK and South African programs is the introduction of legislation in South Africa. Whilst the voluntary approach of the UK is undoubtedly having an impact, some experts claim that voluntary processes are unlikely to be sustainable for most countries and that legislation is required [80]. Whilst legislation may take longer to introduce and be less flexible, it helps to rapidly create a level playing field for food manufacturers and is more likely to survive leadership changes [80].

The introduction of legislation on salt targets in foods was driven by an urgent need in South Africa to address the increasing burden of disease related to chronic diseases. Unlike the UK, where communicable diseases are fairly well controlled, South Africa faces a quadruple burden of disease, which includes, HIV and tuberculosis, and high maternal and infant mortality, in addition to non-communicable diseases and injuries [81]. In the sub-Saharan African region, it is estimated that if the 10–20 million people believed to have hypertension were treated, about a quarter of a million deaths and twice as many long-term disabilities would be prevented annually [82].

Micronutrient deficiencies still exist in some sectors of the population in South Africa. Since 1995, universal salt iodisation (USI) has successfully eradicated iodine deficiency in the country [83], with subsequent concerns that reductions in use of discretionary salt may jeopardize the gains realized by the USI programme. Some evidence exists that salt intake targets are compatible with adequate iodine status in countries with USI. A South African study has reported that consumers with salt intakes within the recommended range of <5 g/day are iodine replete and that median urinary iodine concentrations did not differ across categories of salt intake [84]. In the absence of USI in the UK, salt reduction will not impact on iodine status, but recent evidence suggests that sub-optimal intakes of iodine, particularly in teenage girls [85], needs to be addressed through other nutritional interventions. The WHO [86] recommends that ongoing surveillance of iodine status is required as salt intakes of populations drop, in order for the amount of iodine added as a fortificant to iodised salt to be increased, as necessary, without risk of excess [86]. In addition, the communication activities of salt reduction and iodine deficiency elimination programs need to be effectively co-ordinated to ensure that the potential health benefits of both programs are maximized [86].

## 4. Conclusions

As with all public health issues, translation from research to policy requires advocacy and lobbying from respected coalitions, along with strong governmental leadership. Variations on this process were followed in both the UK and South Africa for the implementation of their salt reduction campaigns. It remains to be demonstrated whether mandatory legislation is more effective than voluntary target-setting in reducing population-level salt intake. Ongoing monitoring of the food supply in both of these countries is essential to measure impact, while data on trends in health outcomes is needed to inform outcome evaluation. The experiences from these two countries provide guidance for other countries that are in the process of developing salt reduction strategies to address the burden of cardiovascular disease.
